# CellTICS: an explainable neural network for cell-type identification and interpretation based on single-cell RNA-seq data

**DOI:** 10.1093/bib/bbad449

**Published:** 2023-12-07

**Authors:** Qingyang Yin, Liang Chen

**Affiliations:** Department of Quantitative and Computational Biology, University of Southern California, 1050 Childs Way, Los Angeles, CA 90089, United States; Department of Quantitative and Computational Biology, University of Southern California, 1050 Childs Way, Los Angeles, CA 90089, United States

**Keywords:** explainable neural network, cell type identification, pathways, scRNA-seq data

## Abstract

Identifying cell types is crucial for understanding the functional units of an organism. Machine learning has shown promising performance in identifying cell types, but many existing methods lack biological significance due to poor interpretability. However, it is of the utmost importance to understand what makes cells share the same function and form a specific cell type, motivating us to propose a biologically interpretable method. CellTICS prioritizes marker genes with cell-type-specific expression, using a hierarchy of biological pathways for neural network construction, and applying a multi-predictive-layer strategy to predict cell and sub-cell types. CellTICS usually outperforms existing methods in prediction accuracy. Moreover, CellTICS can reveal pathways that define a cell type or a cell type under specific physiological conditions, such as disease or aging. The nonlinear nature of neural networks enables us to identify many novel pathways. Interestingly, some of the pathways identified by CellTICS exhibit differential expression “variability” rather than differential expression across cell types, indicating that expression stochasticity within a pathway could be an important feature characteristic of a cell type. Overall, CellTICS provides a biologically interpretable method for identifying and characterizing cell types, shedding light on the underlying pathways that define cellular heterogeneity and its role in organismal function. CellTICS is available at https://github.com/qyyin0516/CellTICS.

## INTRODUCTION

Single-cell RNA-sequencing (scRNA-seq) provides expression profiles of individual cells and molecularly defines cell states or phenotypes [1]. It also has the potential to identify cell types, which are functional units of an organism and crucial to other downstream research investigations. Recently, machine learning has been widely applied to identifying cell types. Typically, a machine-learning model is built using a reference dataset with well-curated cell-type labels. Then, the model is applied to the query dataset to predict its cell types. Deep learning has consistently shown promising performances in cell-type identification for various architectures and the customization of hyperparameters [2]. Numerous neural network architectures have contributed to these achievements, including artificial neural networks [3, 4], convolutional neural networks [5, 6], graph-based networks [7, 8], generative models (autoencoders or generative adversarial networks) [9–11] and attention-based models (transformers) [12, 13]. Moreover, recent advances in multimodal learning show its immense potential in bioinformatics [14, 15]. However, these deep learning-based models are usually black-box models, lacking interpretability. Moreover, predicting at the sub-cell-type level has always been more challenging, even though it provides higher resolution and can unveil valuable insights into cell heterogeneity [16]. Motivated by existing limitations, this study aims to address both the aspects of biological interpretability and sub-cell-level prediction.

In the field of computational biology, interpretable neural networks are constructed using prior biological knowledge, such as gene ontology and pathway information, to improve the interpretability of deep learning. For instance, DrugCell [17] uses a visible neural network that predicts anti-cancer drug responses by leveraging biological process information from the Gene Ontology (GO) database [18]. P-NET [19] is a biologically informed deep-learning model for prostate cancer discovery. Liu et al. [20] introduced an interpretable deep neural network that faithfully captures the underlying chemical mechanism of transcription factor binding to DNA. CancerIDP [21] is an interpretable neural network that predicts cancer patients’ survival based on drug prescriptions and personal transcriptomes. In these models, neurons are imbued with biological meaning (GO terms, pathways, etc.), and certain automatic computational procedures, like DeepLIFT [22], are employed to evaluate the significance of these neurons. Hence, these models significantly enhance human understanding of hidden mechanisms [23] and have shown remarkable success in both predicting classification labels and explaining results. Nevertheless, there are few studies that focus on the application of interpretable neural networks for cell type annotation and the interpretation of the factors that define a cell type.

Inspired by the success of interpretable neural networks, this study presents **CellTICS** (**Cell**-**T**ype **I**dentifi**C**ation based on **S**cRNA-seq), a biologically explainable neural network for cell-type identification and interpretation based on single-cell RNA-seq data. In CellTICS, marker genes are prioritized as interpretable features. The network architecture of CellTICS follows the hierarchy of pathways acquired from the Reactome database [24] and adopts a two-stage strategy to predict cell and sub-cell types. What sets CellTICS apart is its innovative approach of utilizing multiple prediction layers to aggregate information from various pathway levels. This unique multi-predictive-layer strategy greatly enhances the accuracy of the pathway-guided encoder’s predictions. The superior classification performance of CellTICS is demonstrated by applying it to several public datasets and comparing it with other cell-type annotation methods. More importantly, the interpretability of CellTICS provides clues about which pathway characteristics are essential to define a cell type or a cell type under a specific physiological condition. This information is often missed by traditional methods, making CellTICS a valuable tool for understanding the biology of cellular heterogeneity.

The remaining sections of this article are organized as follows. In the “2 Material and methods” section, an overview of the utilized datasets is provided, followed by a detailed explanation of the CellTICS model and its associated parameters, and the description of other methods used for comparison along with the evaluation criteria. In the “3 Results and discussions” section, a comprehensive discussion of the results and innovative aspects of this work is undertaken. It commences with the presentation of cell-type and sub-cell-type annotation results. Following that, CellTICS’s capability to uncover novel pathways and their characteristics related to expression stochasticity are explored. Its application in the context of the aging process and autism spectrum disorder (ASD) is also examined. Finally, in the “4 Conclusion” section, the study is drawn to a close by summarizing the key findings and insights derived from our research.

## MATERIALS AND METHODS

**Figure 1:**
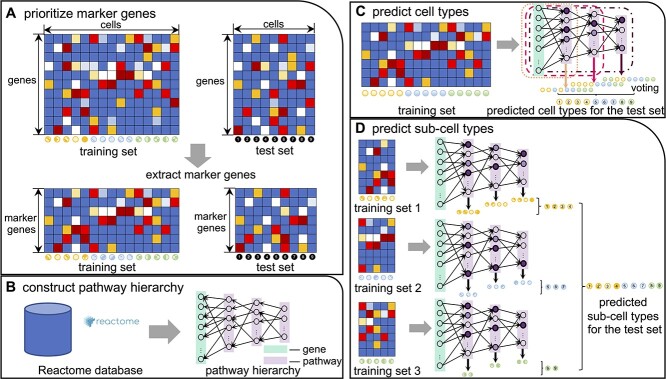
**The pipeline of CellTICS.** (**A**) Marker genes are prioritized by considering their cell-type-specific high and low expression measures. (**B**) The Reactome pathways are used to construct the network hierarchy. (**C**) A neural network constructed by the pathway hierarchy is trained and then used to predict cell types of the test set. The predicted results from all predictive layers are integrated by voting. (**D**) Based on the predicted results of the first stage, several sub-neural networks are trained for sub-cell-type prediction. Important pathways defining a cell type (or a sub-cell type) can be obtained by comparing their activation values across different cell types.

The schematic pipeline of CellTICS is shown in Figure 1. In CellTICS, marker genes are selected based on their expression levels in the training set. The neural network is constructed as the hierarchy of Reactome pathways. A two-stage deep learning strategy is employed to predict both cell types and sub-cell types. In the first stage, a feedforward neural network is trained for cell-type prediction, with a predictive layer corresponding to each pathway hidden layer. Subsequently, in the second stage, multiple sub-neural networks are trained for sub-cell-type prediction, building upon the predicted outcomes from the first stage. Each sub-neural network is also equipped with multiple predictive layers for hidden layers. At each stage, prediction results from all predictive layers are aggregated through a voting mechanism. Furthermore, during the training procedure, important pathways that contribute to (sub-)cell-type prediction can be identified by comparing the activation values of neural networks corresponding to cells belonging to different cell types. More details are as follows.

### Datasets

The capabilities of CellTICS were showcased using scRNA-seq data from three mouse brain studies [25–27] and two human studies [28, 29]. Its predictive accuracy was evaluated across a total of 10 analysis tasks, including both intra-dataset and inter-dataset predictions (Supplementary Table S1). The entire process of the data processing and handling is illustrated in Supplementary Figure S1.

#### Raw scRNA-seq data processing

The detailed datasets used in this study included the level-5 mouse brain data (L5MB), DropViz hippocampus (DropViz HC), DropViz frontal cortex (DropViz FC), and human peripheral blood mononuclear (PBMC) data, all of which were initially in raw read count format. In contrast, the aging mouse brain (AMB) and autism spectrum disorder (ASD) data were first scaled to 10,000 transcripts per cell and then subjected to logarithmically transformed. As a result, a normalization step was necessary for the L5MB, DropViz HC, DropViz FC, and PBMC data.

Let us denote $expr_{c,g}$ as the normalized expression measure for gene $g$ in cell $c$. The normalization is performed as Equation 1, where $m_{c,g}$ is the gene-level read count (UMI-based), and $G$ is the total number of genes. Subsequently, quality control for all data was performed by the R package scater [30], filtering out cells and genes with poor quality. 


(1) \begin{align*}& expr_{c,g}=log_{2}\left(10000\frac{m_{c,g}}{\sum_{g^{\prime}=1}^{G}m_{c,g^{\prime}}}+1\right)\end{align*}

#### Training sets and test sets preparation

The L5MB, DropViz HC and DropViz FC datasets contained information on cell types and sub-cell types. To represent intra-dataset predictions, these datasets were split into a training set and a test set using the R package caret [31] with 75% of the original data allocated to the training set, stratified by cell types.

The AMB data contained mouse brain cells for the young and old age groups as well as their cell types and sub-cell types. We created three analysis tasks based on the data. The first one was the original whole dataset (AMB whole), incorporating age status into the cell-type labels to create our own sub-cell-type labels (e.g. old-cell-type-A versus young-cell-type-A). Next, the AMB whole dataset was split into a training set and a test set with 75% of the original data allocated to the training set, stratified by cell types. The other two analysis tasks were designed for inter-dataset prediction for both cell types and original sub-cell types. Specifically, the old data were treated as the training set and the young data as the test set (AMB OY), or vice versa (AMB YO).

The PBMC data were sequenced by the 10X Genomics v2 (10Xv2), the 10X Genomics v3 (10Xv3) and the Drop-seq protocols. The three major cell type groups were lymphoid cells (including sub-cell types: B cells, CD4 T cells, cytotoxic T cells and natural killer cells), myeloid cells (including sub-cell types: CD14+ monocytes, CD16+ monocytes, dendritic cells and plasmacytoid dendritic cells) and megakaryocytes [32]. Based on the PBMC data, three analysis tasks were created to test the cross-protocol prediction, a special case of inter-dataset prediction. Firstly, the 10Xv3 data were used as the training set, and the 10Xv2 data were the test set (PBMC 32). Secondly, the 10Xv2 data were the training set, and the Drop-seq data were the test set (PBMC 2D). Lastly, the Drop-seq data were the training set, and the 10Xv3 data were the test set (PBMC D3).

The ASD data contained human brain data for the ASD and control groups. To explore the mechanism of ASD at the cell-type level, this work incorporated the disease status into the cell-type labels to create the sub-cell-type labels (e.g. ASD-cell-type-A versus control-cell-type-A). This study focused exclusively on 11 neuron cell types, namely L2/3, L4, L5/6, L5/6-CC, IN-PV, IN-SST, IN-SV2C, IN-VIP, Neu-mat, Neu-NRGN-I and Neu-NRGN-II. Subsequently, the dataset was split into a training set and a test set with 75% of the original data allocated to the training set, stratified by cell types. The processed data were written into CSV files and uploaded to Zenodo (see ‘Code and Data Availability’). The CSV file sizes have also been included in Supplementary Table S1.

### The CellTICS model

#### Marker gene prioritization

A feature selection step was performed to prioritize marker genes, which could help enhance the prediction performance of the CellTICS model. The cell-type-specific high expression measure for a gene $g$ in cell type $t$ is defined as Equation 2, where $\tau _{g}$, $\hat{x}_{t,g}$ are determined by Equation 3 and Equation 4. Here, $n$ is the total number of cell types, and $expr_{t,g}$ is the normalized expression measure for the gene in cell type $t$. Pseudo number 1 is added to avoid extremely large fold changes among lowly expressed genes. Thus, $0\leq \varphi _{t,g}\leq 1$. The cell-type-specificity measure is a modification of the tissue-specificity measure proposed by Jiang et al. [33]. A larger $\varphi _{t,g}$ value suggests that the gene is specifically highly expressed in this cell type. For the cell type with the maximum expression, $\varphi _{t,g}=\tau _{g}$, otherwise $\varphi _{t,g}<\tau _{g}$. 


(2) \begin{align*} & \varphi_{t,g}=\tau_{g}\hat{x}_{t,g} \end{align*}

(3) \begin{align*} & \tau_{g}=\frac{\sum_{t=1}^{n}(1-\hat{x}_{t,g})}{n-1} \end{align*}

(4) \begin{align*} & \hat{x}_{t,g}=\frac{expr_{t,g}+1}{\max_{1\leq t^{\prime}\leq n}expr_{t^{\prime},g}+1} \end{align*}

Similarly, the cell-type-specific low expression measure for a gene $g$ in cell type $t$ is defined as Equation 5, where $\omega _{g}$, $\hat{z}_{t,g}$ are determined by Equation 6 and Equation 7. Thus, $0\leq \rho _{t,g}\leq 1$. A larger $\rho _{t,g}$ value suggests that the gene is specifically lowly expressed in this cell type. 


(5) \begin{align*} & \rho_{t,g}=\omega_{g}\hat{z}_{t,g} \end{align*}

(6) \begin{align*} & \omega_{g}=\frac{\sum_{t=1}^{n}(1-\hat{z}_{t,g})}{n-1} \end{align*}

(7) \begin{align*} & \hat{z}_{t,g}=\frac{\min_{1\leq t^{\prime}\leq n}expr_{t^{\prime},g}+1}{expr_{t,g}+1} \end{align*}

To select cell-type-specific highly expressed or lowly expressed genes, for a gene $g$ in cell type $t$, if $\varphi _{t,g}$ was greater than the $100\alpha $th quantile of $\varphi $ in cell type $t$, the gene would be selected as a marker gene for the cell type. Similarly, for another gene $g^{\prime}$ in cell type $t^{\prime}$, if $\rho _{t^{\prime},g^{\prime}}$ was greater than the $100\beta $th quantile of $\rho $ in cell type $t^{\prime}$, the gene would be also selected as a marker gene for the cell type. All marker genes from all cell types were combined as the result of the feature selection step. These marker genes were used to compress both the training set and the test set.

#### Pathway hierarchy

All levels of the pathway hierarchy were acquired from the Reactome database, which could be represented as a tree. The pathway hierarchies for mice, humans, and rats were available in CellTICS. The tree was composed of internal nodes representing hierarchical pathways and leaves representing genes. Pathways at the highest level were connected to the root node, and edges and nodes not related to the selected marker genes were excluded. To formally formulate the network, the parameter $l$ was introduced to specify the number of considered levels (i.e. the number of hidden layers of the neural network). When $l$ was specified, a subtree of the original tree could be constructed. If the upper depth of a leaf was larger than $l$, the excessive parental level(s) of the leave would be excluded and the leaf would be connected to upper-level pathways. If the upper depth of a leaf was smaller than $l$, artificial nodes would be added to represent the parents of the leaf. From the subtree, a binary mask matrix $M$ was generated to suggest the connectivity of two entities in adjacent layers. If the two entities had no connection, the corresponding entry in $M$ was 0; otherwise, it would be 1.

#### Neural network construction

A feedforward neural network was constructed based on the mask matrix. We first trained the network for cell-type prediction and then trained several sub-neural networks for sub-cell-type prediction. Thus, after obtaining the predicted cell types for the test set, the test set was divided into subsets based on these predictions, and similarly, the training set was separated based on the true cell types. Then, sub-cell-type prediction was performed for each cell type.

The detailed architecture of the CellTICS neural network is shown in Supplementary Figure S2. In the neural network, each node represents an entity such as a gene, pathway, or cell type, and each edge represents the relationship between the entities connected by the edge. The input layer comprises a set of genes. The input data are the expression level of these genes, whose dimension is the number of genes assigned to the bottom pathway level. For each layer, the input is denoted as $x$ and the output is denoted as $y$. The output of the layer is calculated using Equation 8, where $f$ is the activation function, $M$ is the mask matrix, $W$ is the weight matrix, and $b$ is the bias vector. Here $MW$ is the element-wise multiplication of $M$ and $W$ (i.e. Hadamard product). The activation function is the tanh function (Equation 9). The activation values are related to the importance measures of pathways, and they will be elaborated upon in 2.2.4. The dimension of each hidden layer is in accordance with the mask matrix $M$. To obtain the prediction for each cell, a predictive layer is added after each hidden layer. The dimension of each prediction layer equals the number of cell types. The probability of a cell being classified as the $i$th cell type is calculated using the softmax function (Equation 10), where $C$ is the total number of cell types and $z$ is the input of the softmax function. The cell is assigned to the class with the highest probability. Finally, the predictions from all predictive layers are integrated by voting to obtain the final prediction result. 


(8) \begin{align*} & y=f[(MW)x+b] \end{align*}

(9) \begin{align*} & f=tanh(z)=\frac{e^{z}-e^{-z}}{e^{z}+e^{-z}} \end{align*}

(10) \begin{align*} & \sigma_{i}=\frac{e^{z_{i}}}{\sum_{c=1}^{C}e^{z_{c}}} \end{align*}

#### Important pathway identification

To identify important pathways for distinguishing each (sub-) cell type, CellTICS utilized activation values during the training procedure. Specifically, the activation values were calculated for each pathway in every layer in the CellTICS neural network. For a given pathway $p$ and cell type $t$, the average activation value ($A_{t,p}$) was computed across all cells of that type. These average activation values were then sorted in descending order across all cell types, obtaining a list ($A_{(1),p}$,$A_{(2),p}$,...,$A_{(T),p}$), where $T$ is the total number of cell types. To quantify the importance of pathway $p$, the difference between the first and second largest average activation values, Equation 11, was computed. Due to the possibility of a pathway $p$ being included in the paths of multiple prediction layers, there might be several $d_{p}$ values. For instance, a pathway located on the first hidden layer (level 1) could be present in the network paths leading to prediction level 1, level 2, and so on, until the final hidden layer was reached. Hence, the maximum value of $d_{p}$ among all paths of such nested networks was selected as the final importance measure of the pathway and denoted as $D_{p}$. If the importance value $D_{p}$ exceeded a predefined threshold (default: 0.1), pathway $p$ would be deemed important for the cell type corresponding to $A_{(1),p}$. 


(11) \begin{align*}& d_{p}=A_{(1),p}-A_{(2),p}\end{align*}

### Parameter settings

The CellTICS model was trained by the Adam optimizer with the cross-entropy loss function with L2 regularization. To optimize the performance of the CellTICS model, the parameters were tuned using grid search with 5-fold cross-validation. The default values for threshold values ($\alpha $ and $\beta $) are 0.95 and 0.9, respectively. The default value for the number of hidden layers of the neural network ($l$) is 5. The default hyperparameters for the minibatch size, learning rate, and regularization parameter are 32, 0.001, and 0.0001, respectively. The default number of epochs is 10 for cell-type prediction and 50 for sub-cell-type prediction.

### Method comparison

This work compared the performance of CellTICS with six other methods to evaluate its predictive performance. The compared methods were SingleR [34], scmap [35] (containing scmap-cluster and scmap-cell), CHETAH [36], scPred [37], and ACTINN [3]. We selected these specific models based on their representation of major categories within cell type annotation methods, encompassing correlation-based methods (SingleR, scmap, and CHETAH), supervised classification-based methods (scPred), and traditional neural networks (ACTINN) [38]. Noteworthy, we compared CellTICS with ACTINN, a traditional neural network-based method. This choice was made to assess CellTICS’s proficiency in accurate cell classification and check whether its interpretability sacrifices the prediction accuracy. The default parameter settings of these tools were used unless mentioned otherwise. For scmap, the unassigned threshold, which represents a similarity threshold, was set to $-1$.

### Evaluation criteria

#### Evaluation of cell type prediction

The performance of CellTICS and the compared methods were evaluated using two metrics: accuracy (ACC) and macro-averaged F1 scores (macro F1 scores). ACC measures the percentage of cells correctly classified, while the F1 score is the harmonic mean of precision and recall. The macro F1 score is computed by taking the arithmetic mean of all per-class F1 scores and is useful when dealing with class imbalance problems, which are common in cell-type classification.

#### Evaluation of important pathway identification

To demonstrate the unique advantages of CellTICS in identifying cell-type important pathways, the gene set enrichment analysis (GSEA) [39] was utilized for comparison. GSEA is a method for evaluating cumulative changes in the expression of multiple genes belonging to a gene set (i.e. a pathway). It can be used to identify pathways exhibiting differential gene expression between cell types, between the young and old cells of a specific cell type, or between diseased and healthy cells of a cell type.

The logarithmic fold change (logFC) was calculated as a differential expression measure for a gene by computing the difference of average log-normalized count data in different groups. Subsequently, the R package ReactomePA [40] was used to perform GSEA and identify important pathways with a *P*-value less than 0.05. Finally, the important pathways found by CellTICS and GSEA were compared.

For pathways identified by CellTICS but not by GSEA, Wilcoxon signed-rank tests were further performed to examine the variation difference, instead of the traditional mean difference, between groups. Thus, for each important pathway identified by CellTICS, the coefficient of variation (CV) of all genes in both groups (e.g. cells of cell type A versus other cells, or old cells of cell type A versus young cells of cell type A) was calculated. Consequently, a Wilcoxon signed-rank test on the CV values of the two groups was performed to assess the significance of variation differences.

## RESULTS AND DISCUSSIONS

### Identification of cell and sub-cell types

To illustrate the predictive power of CellTICS for (sub-)cell-type identification, we compared CellTICS with six other methods for 10 analysis tasks (Supplementary Table S1). The procedure was repeated five times across all datasets, and the average ACC and macro F1 scores were computed.

**Figure 2:**
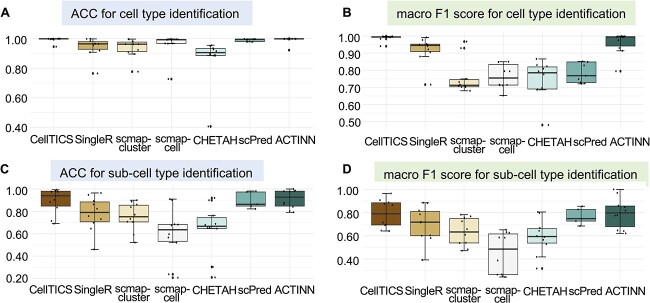
**The prediction performance of CellTICS and other cell-type identification methods.** A total of 10 analysis tasks were conducted to generate the boxplots. In each of these tasks, the average ACC and macro F1 scores based on five analysis repeats were used. (**A**) The ACC for cell-type identification. (**B**) The macro F1 score for cell-type identification. (**C**) The ACC for sub-cell-type identification. (**D**) The macro F1 score for sub-cell-type identification.

The results for the cell-type identification and the sub-cell-type identification are shown in Figure 2. Clearly, CellTICS outperforms the other methods in almost all datasets, since the performance of CellTICS is usually the best, that is, exhibiting higher scores and smaller performance variations. Although most methods can obtain high ACC for major cell type identification (Figure 2A), the sensitivity of many methods is not satisfactory enough as reflected by the low macro F1 scores in Figure 2B. However, CellTICS stands out with an average macro F1 score between 0.9406 and 0.9993 for the considered datasets. Classifying sub-cell types is a more challenging task, while CellTICS is still superior in most methods (Figure 2C and Figure 2D). The performance of these models for each analysis task is illustrated in Supplementary Figure S3.

We also compared CellTICS with the original marker gene selection to CellTICS with highly variable gene (HVG) selection (top 2,000, 5,000, or 10,000 HVGs). As shown in Supplementary Figure S4, the performance of original CellTICS is significantly better than using the top 2,000 HVGs, while it is comparable with using the top 5,000 and 10,000 HVGs. In the ASD data analysis, CellTICS’s marker gene prioritization performs much better than the HVG approaches (Supplementary Figures S4A–D).

CellTICS is robust in cell type and sub-cell type identification. We ran CellTICS 20 times and observed very small variations in the accuracy and macro F1 scores for all cell type prediction and sub-cell type prediction (Supplementary Figure S5). Also, CellTICS is computationally efficient. As shown in Supplementary Table S2, for the L5MB dataset which included about 20,000 cells and about 28,000 genes, the whole runtime (including training and testing) was about 23 min.

**Figure 3:**
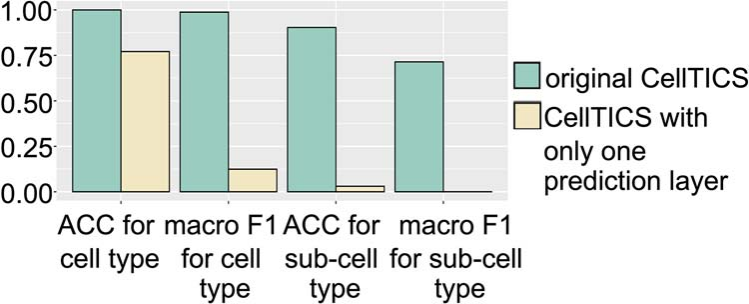
**The importance of the multi-predictive-layer strategy.** The average ACC and macro F1 scores of the original CellTICS and CellTICS with only one prediction layer are shown for cell-type and sub-cell-type identification on the L5MB dataset.

One major distinctive part of CellTICS is the multi-predictive-layer strategy, which greatly improves the prediction accuracy and prediction power. Figure 3 demonstrates the effectiveness of this strategy by comparing the performance of the original CellTICS with that of CellTICS containing only one prediction layer on the L5MB dataset. The results show that multiple prediction layers can significantly enhance the ACC and macro F1 score of the predictions. For cell type classification, the ACC is improved from 0.7711 to 0.9997, while the macro F1 score is improved from 0.1244 to 0.9878. In sub-cell type classification, the improvement is even more significant, with the ACC increasing from 0.0303 to 0.9035 and the macro F1 score increasing from 0.0003 to 0.7142. These findings demonstrate that the multi-predictive-layer strategy adopted by CellTICS can substantially improve the accuracy of predictions, providing a more nuanced and comprehensive understanding of cell heterogeneity and disease processes. The key advantage of this approach is that it allows for the aggregation of information from different levels of pathway hierarchy, particularly those at the bottom levels which represent more detailed pathways. By leveraging the unique information content available at each level, the network can capture both the broad functional relationships between pathways and the more specific functional roles played by individual pathways.

Another noteworthy property of CellTICS is its ability to make predictions at the sub-cell-type level. CellTICS outperforms most methods in most cases (Supplementary Figures S3C–D), even for the L5MB dataset, where the number of sub-cell types is relatively large (i.e. 220). It also implies that CellTICS has the potential to handle the class imbalance problem since the percentage of sub-cell types in the L5MB test set varies from 0.01 to 5.01%. The success in predicting sub-cell types suggests that CellTICS can better facilitate the construction of cell atlas in complex tissues to a deeper level. At this level, the identification of rare cell types might hold special significance [41]. Rare cell types are sporadic within a large population of cells, and their marker genes may exhibit very weak signals. However, CellTICS can capture these signals by incorporating both highly and lowly expressed marker genes of all cell types. Particularly, the inclusion of cell-type-specific lowly expressed genes offers valuable insights as their expression remains uniquely low in certain cell types, which is often overlooked in traditional marker gene prioritization. The comparison between CellTICS’s marker genes and HVGs supports this assertion.

The trade-off between interpretability and prediction accuracy for deep neural networks is a common challenge in machine learning. As the complexity of the model increases to achieve higher accuracy, its interpretability tends to decrease. However, the hidden layers of CellTICS follow the pathway hierarchy and reflect the real relationships between pathway nodes in a cell. The above results and discussions show that CellTICS can achieve high prediction accuracy. In the following sections, CellTICS’s interpretability will be demonstrated.

### CellTICS identifies important pathways missed by GSEA

Many deep learning models designed for cell type annotation lack the ability to uncover what defines a cell type, which has prompted us to emphasize achieving biological interpretability. In addition to predicting cell types accurately and identifying the genes that contribute to the prediction (i.e. important features), CellTICS can also pinpoint important pathways (i.e. important neurons in the neural network architecture) that define (sub-) cell types or cell types under a specific physiological condition. This capability represents the most distinctive aspect of this study. Traditionally GSEA is typically used to identify pathways with enriched gene expression changes between cell types. In this section, the comparison between the two approaches will be shown.

**Figure 4:**
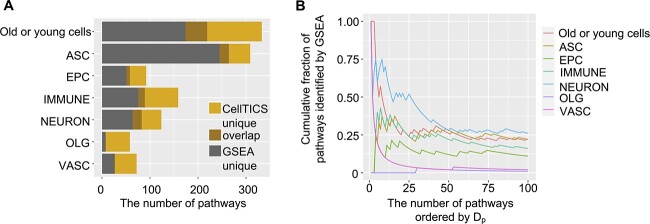
**Comparison between CellTICS and GSEA.** (**A**) The number of important pathways distinguishing cell types or age groups identified by CellTICS ($D_{p}\geq 0.1$) or GSEA. Pathways important for either old or young cells across all cell types are combined for the “Old or young cells” category. (**B**) Shared pathways between CellTICS and GSEA. The cumulative fraction of pathways identified by GSEA is plotted along pathways with decreasing $D_{p}$ values in CellTICS.

For the AMB whole dataset, we compared the important pathways of each cell type identified by CellTICS with those by GSEA and also compared the results for distinguishing old cells from young cells of a specific cell type. As shown in Figure 4A, CellTICS and GSEA share some important pathways for both cell type classification and aging status classification. Moreover, CellTICS discovers many important pathways missed by GSEA, as it can identify nonlinear characteristics of the pathway and capture potential interactions among genes. Some pathways identified by GSEA are not essential in defining cell types, suggesting that the simple add-up of differential signals of genes in a pathway is not necessarily an important cell-type-specific characteristic.

Generally, pathways with larger importance scores of CellTICS ($D_{p}$ values) are more likely to be identified by GSEA (Figure 4B and Supplementary Figure S6). In Figure 4B, pathways from CellTICS are sorted by $D_{p}$ in descending order, and the cumulative fraction of pathways shared between CellTICS and GSEA is shown. The overlapping fraction is generally higher for pathways with higher $D_{p}$ values. Although the number of pathways passing the $D_{p}$ threshold of 0.1 is small for VASC (vascular), OLG (oligodendrocyte), and EPC (ependymocyte) cells (Figure 4A), the pattern still holds for these cell types when ordering pathways by $D_{p}$ without imposing a cutoff (Figure 4B). Alternatively, the $D_{p}$ threshold was adjusted from 0.1 to 0.2 and 0.3 for CellTICS and then compared the results with GSEA. As shown in Supplementary Figure S6, the overlapping fractions are higher when the threshold for CellTICS is more stringent.

The advantage of CellTICS over GSEA is that CellTICS can aggregate differential signals nonlinearly via a neural network approach, while GSEA aggregates the differential signals from individual genes of the same pathway in a linear way. Additionally, CellTICS compares the average activation scores of a pathway between the considered cell type and all other cell types in a pairwise fashion. However, GSEA pools all other cell types together and compares them with the considered cell type.

Knowing pathways that define a (sub-)cell type is particularly valuable because it allows for a better understanding of the molecule dynamics of the cell and how they contribute to predicting a (sub-)cell type. By uncovering cell-type-specific pathways, CellTICS provides a more comprehensive and interpretable view of the underlying biological processes.

### Expression stochasticity of a pathway could be cell-type specific

**Figure 5:**
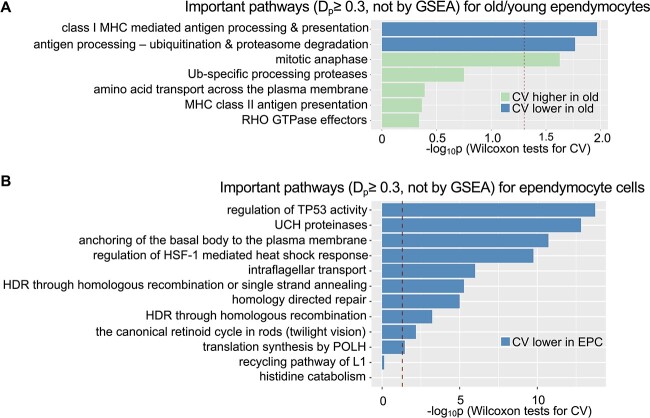
**Important pathways exhibiting differential expression stochasticity.** The differential expression stochasticity of pathways is assessed by Wilcoxon tests for (**A**) different age groups of ependymocytes or (**B**) ependymocytes versus other cell types. Only pathways with $D_{p} \geq 0.3$ in CellTICS but not identified by GSEA are shown. The red dashed lines mark the *P*-value cutoff of 0.05.

As shown in 3.2, some pathways identified by CellTICS do not show significant differential gene expression at the individual gene level and are not identified by GSEA. To explore their underlying mechanisms, we tested the hypothesis that a pathway’s expression stochasticity, measured by the coefficient of variation, could be a cell-type-specific characteristic. For the AMB whole dataset, a Wilcoxon signed-rank test was performed on the coefficient of variation values of two groups (cell type A versus other cell types, or old cells of cell type A versus young cells of cell type A) for the important pathways discovered by CellTICS but not by GSEA. Interestingly, among the eight important pathways (with a $D_{p}$ threshold of 0.3 and not identified by GSEA) for the aging-related pathway discovery, seven of them were discovered in EPCs. Therefore, the differential variation results for pathways related to EPCs are presented in Figure 5. Three out of the seven (Figure 5A) pathways uniquely identified by CellTICS exhibit a significant difference in expression stochasticity between old and young EPCs, and 10 out of 12 (Figure 5B) pathways did so for the comparison between EPCs and other cells (*P*-value $\leq 0.05$, Wilcoxon tests on CVs). Results for other cell types are shown in Supplementary Figures S7–S11, with 14.3–61.1% of their important pathways passing the Wilcoxon tests. These results suggest that expression stochasticity within a pathway could be an essential feature for defining a cell type or a specific age group, which is distinctive for a cell type annotation model. Other studies have also reported that expression stochasticity is important and may affect organism functionality, viability, and flexibility to adapt to environments [42–44].

Based on the findings in Figure 5A, ‘mitotic anaphase’ emerges as one of the most significant important pathways to distinguish age groups of EPCs. According to Hu et al. [45], the anaphase-promoting complex/cyclosome (APC/C) is a novel cellular aging regulator based on its indispensable role in the regulation of lifespan and its involvement in age-associated diseases of eukaryotic organisms. This consistency reinforces the results.

Interestingly, pathways not identified by GSEA but with a higher importance score ($D_{p}$) are usually more likely to exhibit significantly different expression stochasticity (*P*-value $\leq 0.05$, Wilcoxon tests on CVs). As shown in Supplementary Figure S12, approximately 24–79% of the top 100 pathways identified by CellTICS, but overlooked by GSEA, exhibit statistical significance. For most cell types (EPC, NEURON, OLG, VASC, ASC, and old/young cells), there is a decreasing trend in the cumulative overlapping fractions with smaller $D_{p}$ values. The only exception is the IMMUNE cell type. Therefore, for many cell types, expression stochasticity is another important characteristic for a pathway to define the cell type. A similar conclusion can be made when comparing results using different $D_{p}$ thresholds (Supplementary Figure S13).

### Important pathways for cell types undergoing aging

**Figure 6:**
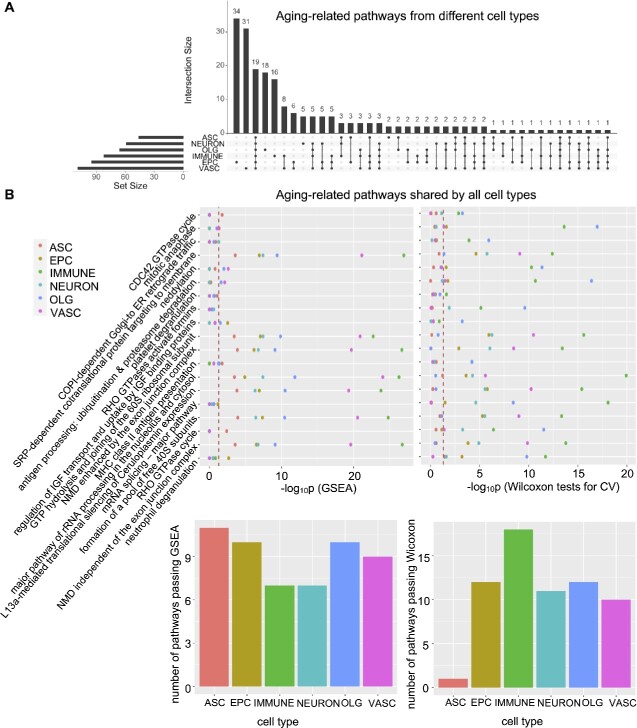
**Aging-related pathways identified by CellTICS.** (**A**) The UpSet plot showing the number of important aging pathways for different cell types ($D_{p} \geq 0.1$). (**B**) GSEA and Wilcoxon tests on CVs for the 19 aging-related pathways shared by all cell types. The corresponding *P*-values are shown. Also, the bar plots show the number of pathways passing GSEA or Wilcoxon tests for each cell type (*P*-values $\leq 0.05$).

Aging-related pathways shown above were based on the two-stage sub-cell-type identification for the AMB whole data. Due to the challenging task of sub-cell-type identification and to achieve more accurate aging-related pathway identification, we carried out the machine learning model for each cell type separately in a single-stage procedure to separate old cells from young cells. Figure 6A shows the UpSet plot of the number of important aging-related pathways ($D_{p} \geq 0.1$) for each cell type. Interestingly, 19 important aging-related pathways are shared by all cell types, suggesting common and essential mechanisms underlying the aging process for all considered cell types.

To investigate the 19 pathways important for aging, both GSEA and the variation-based Wilcoxon signed-rank test were performed. Figure 6B shows that although the 19 pathways are aging-related for all cell types according to CellTICS, differential expression (significant GSEA *P*-values) or the differential expression stochasticity (significant Wilcoxon test *P*-values) can only be detected in a subset of cell types. Notably, these pathways are more likely to exhibit differential expression for astrocytes, while they are more likely to show differential expression stochasticity for immune cells. Although immune cells do not conform to the trend of “larger $D_{p}$, more likely to pass Wilcoxon tests” (Supplementary Figure S12), which means they may not use expression stochasticity to distinguish themselves from other cell types, they utilize it to differentiate old and young cells (Figure 6B).

Furthermore, our work reveals that pathways related to nonsense-mediated mRNA decay (NMD) played an important role in the aging process. NMD is a translation-coupled mechanism that eliminates mRNAs containing premature translation-termination codons [46, 47]. In this analysis, “NMD enhanced by the exon junction complex” and “NMD independent of the exon junction complex” were discovered as important aging pathways. Current experimental research has revealed that RNA surveillance via NMD is crucial for longevity in daf-2/insulin/IGF-1 mutant C. elegans [48]. The computational results in mice can complement this understanding of the role of NMD in the aging process.

### Important pathways for ASD

**Figure 7:**
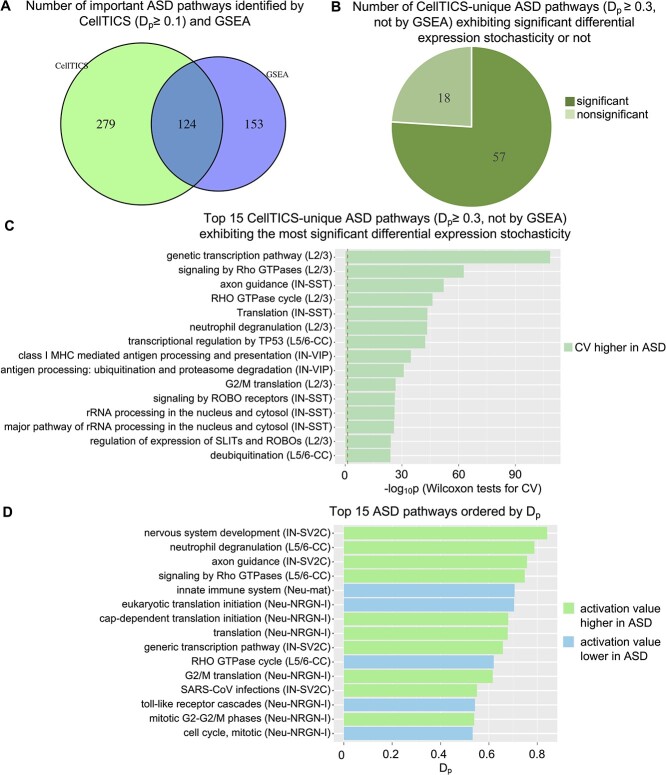
**Important pathways for ASD.** (**A**) The number of important pathways distinguishing disease status identified by CellTICS ($D_{p} \geq 0.1$) or GSEA. (**B**) The number of pathways identified by CellTICS but not by GSEA ($D_{p} \geq 0.3$) with the information whether they pass the Wilcoxon tests on CVs (*P*-values $\leq 0.05$). (**C**) Top 15 CellTICS-unique pathways ($D_{p} \geq 0.3$) ordered by their significance of the Wilcoxon test on CVs. The red dashed line marks the *P*-value of 0.05. The color of each pathway indicates whether the ASD group exhibits a higher or lower coefficient of variation. (**D**) Top 15 important ASD pathways ordered by $D_{p}$ value. Cell types corresponding to each pathway are also shown.

The ASD dataset served as a demonstration of how CellTICS could identify important pathways associated with disease. The results are shown in Figure 7. Similarly, we compared the pathways identified by CellTICS ($D_{p} \geq 0.1$) that distinguish between disease status to those identified by GSEA. Despite some overlaps, CellTICS discovered many more unique pathways (Figure 7A). Then, the $D_{p}$ threshold was tightened to 0.3 and Wilcoxon tests were performed to compare the coefficient of variation of the ASD and the control groups for these CellTICS-unique pathways. The tests show that 57 out of 75 disease pathways exhibit a significant variation difference (Figure 7B), suggesting most of these ASD-related pathways exhibited expression stochasticity.

The top 15 disease pathways ($D_{p} \geq 0.3$, but not identified by GSEA) exhibiting the most significant differential expression stochasticity are shown in Figure 7C, and the top 15 important ASD pathways ordered by $D_{p}$ values are shown in Figure 7D. As expected, the pathway with the highest $D_{p}$ value is “nervous system development”, which is consistent with the etiology of ASD. The analysis reveals that ‘axon guidance’ is among the top three terms with the largest $D_{p}$ value (Figure 7D) and also ranked third for the differential expression stochasticity analysis (Figure 7C). Notably, cortical abnormalities and genetic evidence support the notion that dysregulated axonal growth and guidance are critical developmental processes underlying the clinical manifestations of ASD [49]. The findings further reinforce the importance of axon guidance in ASD.

## CONCLUSION

This study introduces CellTICS, a biologically explainable neural network tailored for cell-type identification and interpretation using single-cell RNA-seq data. CellTICS adopts a two-stage strategy to predict cell types and sub-cell types, and its innovative multi-predictive-layer strategy significantly enhances the prediction accuracy. The prediction accuracy of CellTICS is validated through comparison with alternative methods across various datasets.

Moreover, CellTICS can discover important pathways that are missed by conventional methods like GSEA. These pathways may be characterized by their cell-type-specific expression stochasticity, as quantified by the coefficient of variation in Wilcoxon tests.

Furthermore, the applications of CellTICS in the context of the aging process and ASD underscore its relevance for cell type identification under different physiological conditions. This adaptability is invaluable for gaining insights into how the aging process or specific diseases impact individuals across various cell types. In summary, CellTICS offers a powerful and biologically interpretable tool for advancing our understanding of cellular dynamics and their role in various health and disease states.

**Key Points**

- Innovative Interpretability: CellTICS is significantly different from the traditional ‘black box’ deep learning models, offering biologically interpretable insights into cell type prediction.
- Accuracy Unleashed: With CellTICS, we have pushed the boundaries of accuracy in the identification of cell types and sub-cell types using single-cell RNA sequencing data, providing a sharper lens into the cellular world.
- Pathways Illuminated: CellTICS uncovers the hidden pathways that define unique cell types. It sheds light on the profound influence of expression stochasticity in cell type delineation which is missed by traditional analysis.
- Disease and Aging Unveiled: CellTICS excels at the identification of pathways that set cell types apart under diverse physiological conditions, providing valuable insights into the dynamic changes in cellular landscapes of disease and aging.

## Supplementary Material

supplementary_information_bbad449

## Data Availability

The CSV data underlying this article are available in Zenodo, at https://doi.org/10.5281/zenodo.7829597. Links for accessing the original datasets are available in Supplementary Table S1. Codes for CellTICS and all need libraries information are available at https://github.com/qyyin0516/CellTICS. Specifically, CellTICS was implemented using Python 3 (version 3.9.2), and the libraries involved were numpy (1.23.1), pandas (1.2.3), keras (2.9.0), tensorflow (2.9.0), networkx (2.6.3) and biomart (0.9.2). The computational tasks were executed on a Linux system equipped with two NVIDIA Tesla V100 GPUs and a memory capacity of up to 180 gigabytes. Related pathway findings are also shown on the GitHub page. For data processing and statistical analysis, we employed R (version 4.1.2). Additionally, we used the following R packages: Scater (1.22.0), caret (6.0-93) and ReactomePA (1.38.0). Besides, the HVGs were found using Python package scanpy (1.9.3).

## Author Biographies

**Qingyang Yin** is a Ph.D. student in the Department of Quantitative and Computational Biology at the University of Southern California.

**Liang Chen** is a professor in the Department of Quantitative and Computational Biology at the University of Southern California.
