# Supplementary material for: CellTICS: an explainable neural network for cell-type identification and interpretation based on single-cell RNA-seq data
Source: Brief Bioinform. 2023 Dec 7;25(1):bbad449. doi: 10.1093/bib/bbad449 (PMC10703497; doi:10.1093/bib/bbad449)
Supplement: supplementary_information_bbad449 [file supplementary_information_bbad449.pdf]

Supplementary Information for

CellTICS: an explainable neural network for cell-type identification and interpretation based on single-cell RNA-seq data

Qingyang Yin[1], Liang Chen[1,*]

[1] Department of Quantitative and Computational Biology, University of Southern California, 1050 Childs Way, Los Angeles, CA 90089, United States

[*] To whom correspondence should be addressed. Tel: +1 213-740-2143; Fax: +1 213-821-2506; Email: liang.chen@usc.edu.

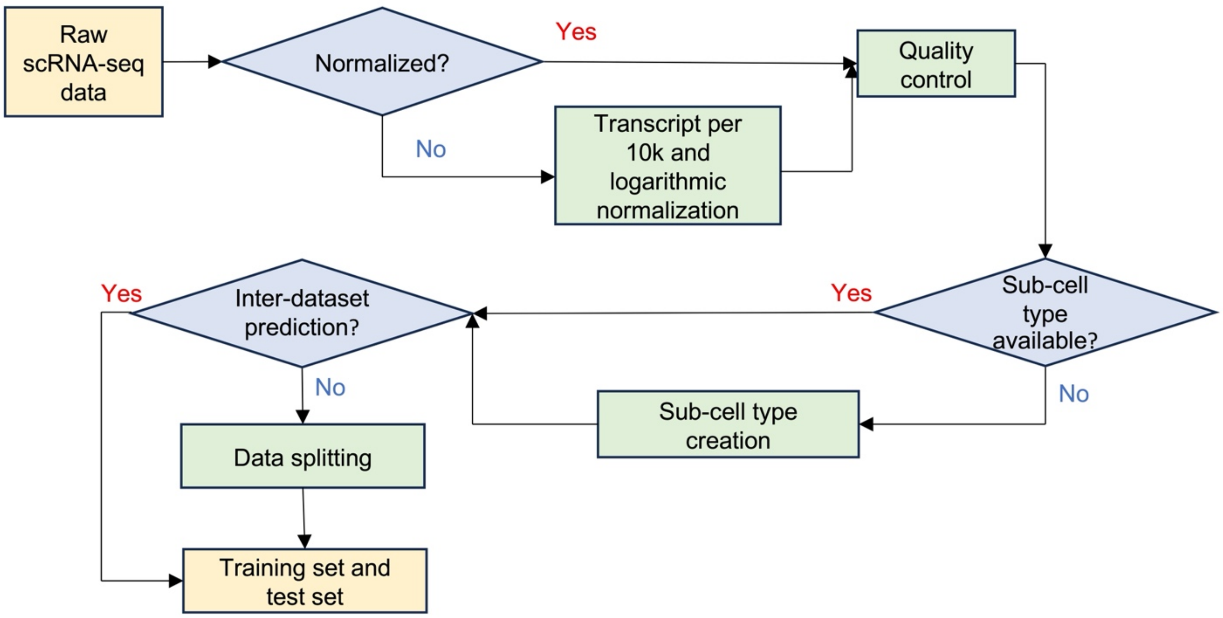

Supplementary Figure S1. Data processing flowchart. Following the acquisition of raw scRNA-seq data, normalization was carried out, contingent upon whether the data had already undergone normalization. Subsequently, after conducting quality control assessments, sub-cell types were generated in cases where they were not initially provided. If the data were not intended for inter-dataset prediction, they were divided into both training and test sets.

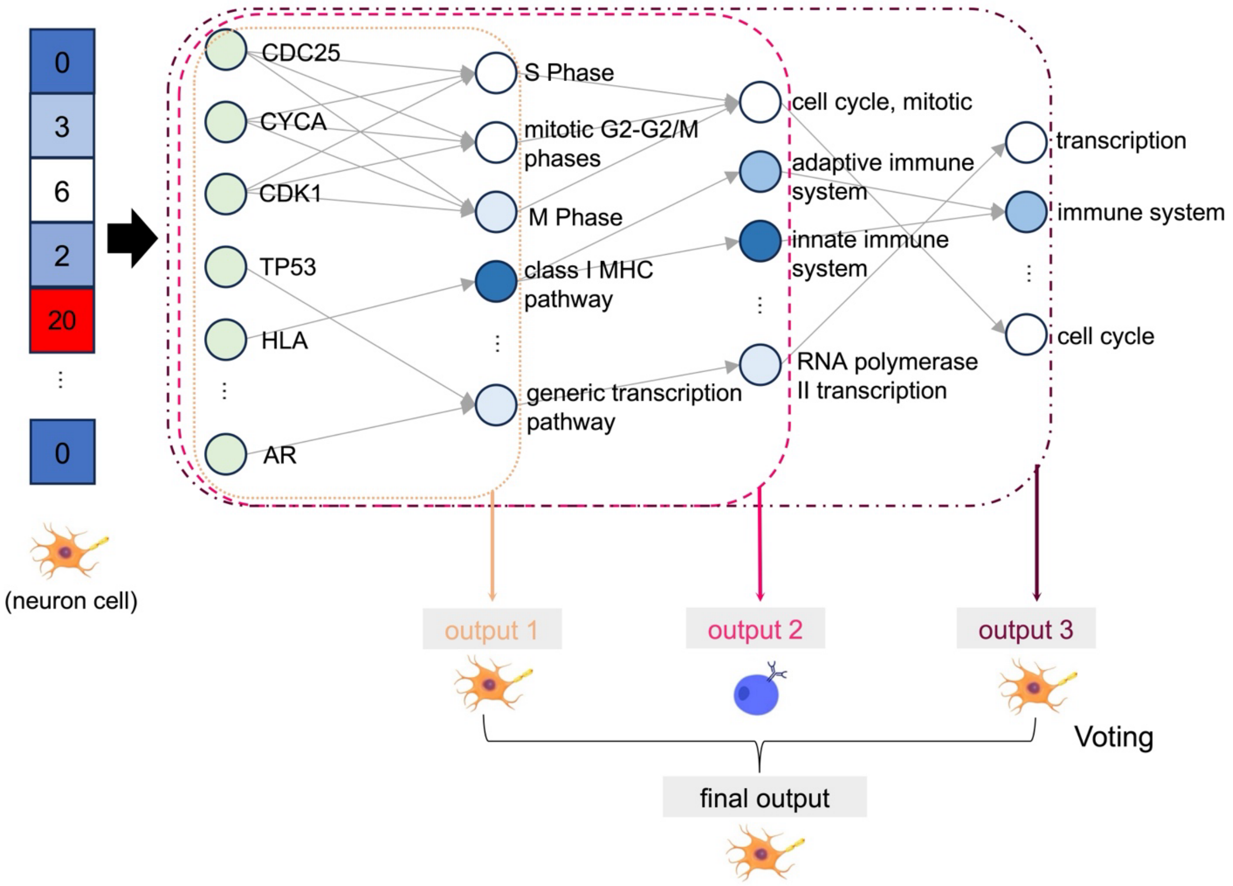

Supplementary Figure S2. The architecture of the CellTICS neural network. In the network, each cell's input comprises the expression levels of all genes within that cell. The input layer corresponds to genes, while the hidden layers model the hierarchical relationships among pathways. After each hidden layer, a predictive layer is introduced to represent cell types. Throughout the training process, significant pathways are identified by harnessing activation values. The predictive results from all predictive layers are then consolidated through a voting mechanism.

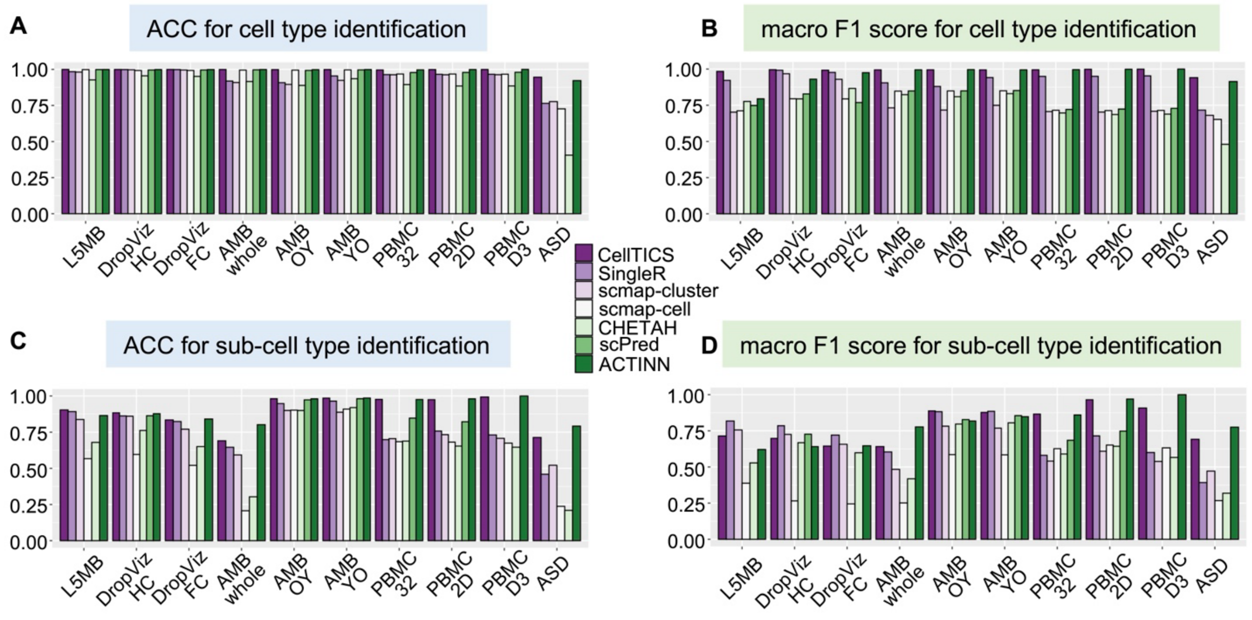

Supplementary Figure S3. The prediction performance of CellTICS and other cell-type identification methods for each analysis task. (A) The ACC for cell-type identification, **(B)** The macro F1 score for cell-type identification, **(C)** The ACC for sub-cell-type identification, **(D)** The macro F1 score for sub-cell-type identification. The ACC and macro F1 scores were average values computed based on five analysis repeats.

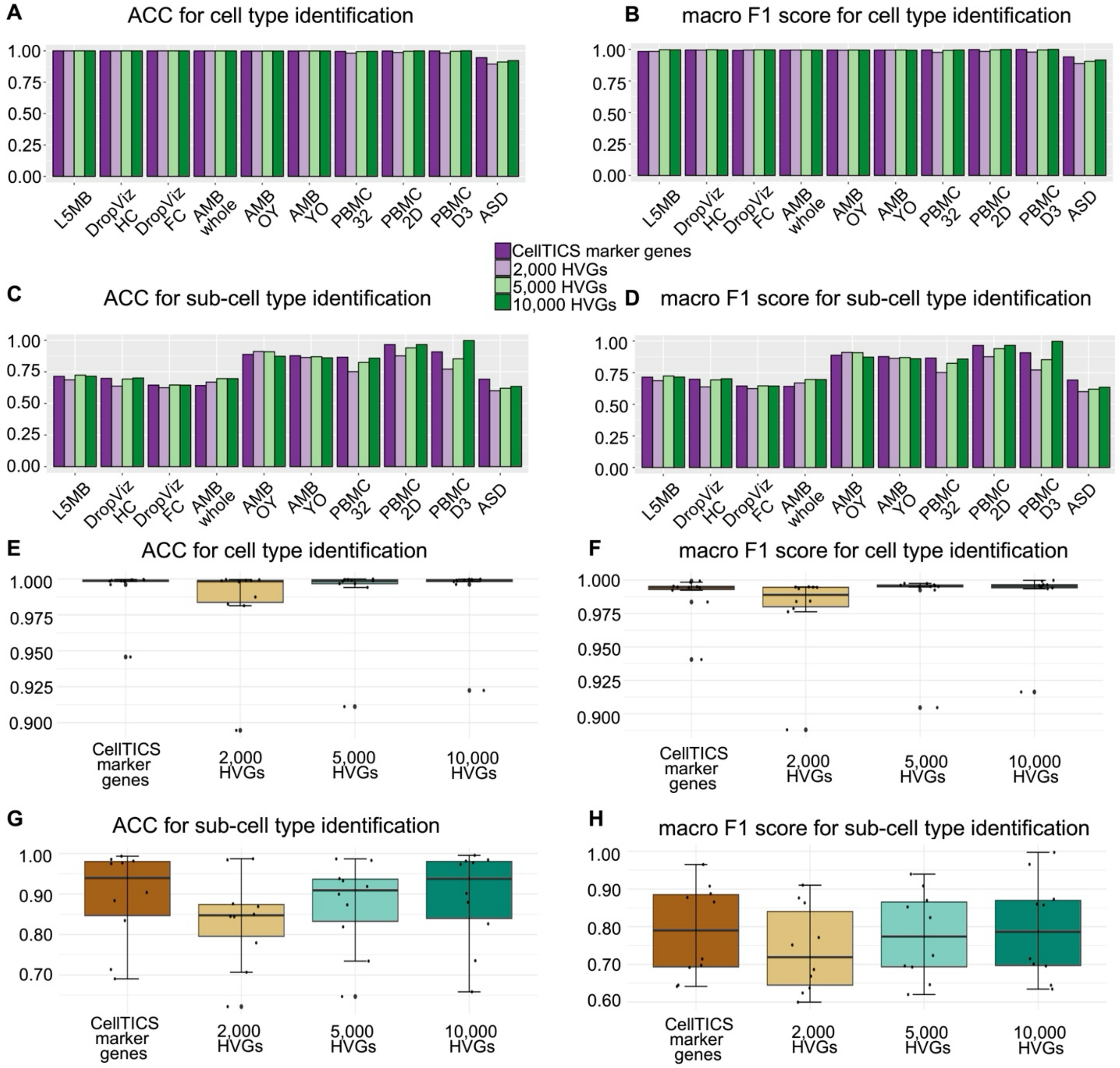

Supplementary Figure S4. Comparison between original CellTICS and CellTICS but using highly variable genes (HVGs). A total of 2000, 5000, or 10000 HVGs were used. A-D are the average values for each dataset. (**A**) The ACC score for cell-type identification. (**B)** The macro F1 score for cell-type identification. (**C**) The ACC score for sub-cell-type identification. (**D**) The macro F1 score for sub-cell-type identification. E-H are boxplots with 10 values for each method. (**E**) The ACC for cell-type identification, (**F**) The macro F1 score for cell-type identification, (**G**) The ACC for sub-cell-type identification, (**H**) The macro F1 score for sub-cell-type identification.

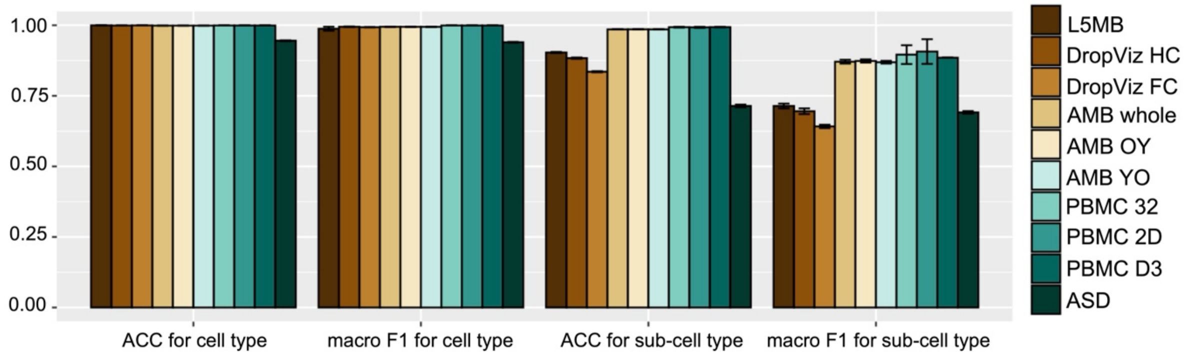

Supplementary Figure S5. Robustness of CellTICS. The average ACC and macro F1 scores of CellTICS for cell-type and sub-cell-type identification across 20 repeats in each dataset are shown. The error bars represent the standard deviation.

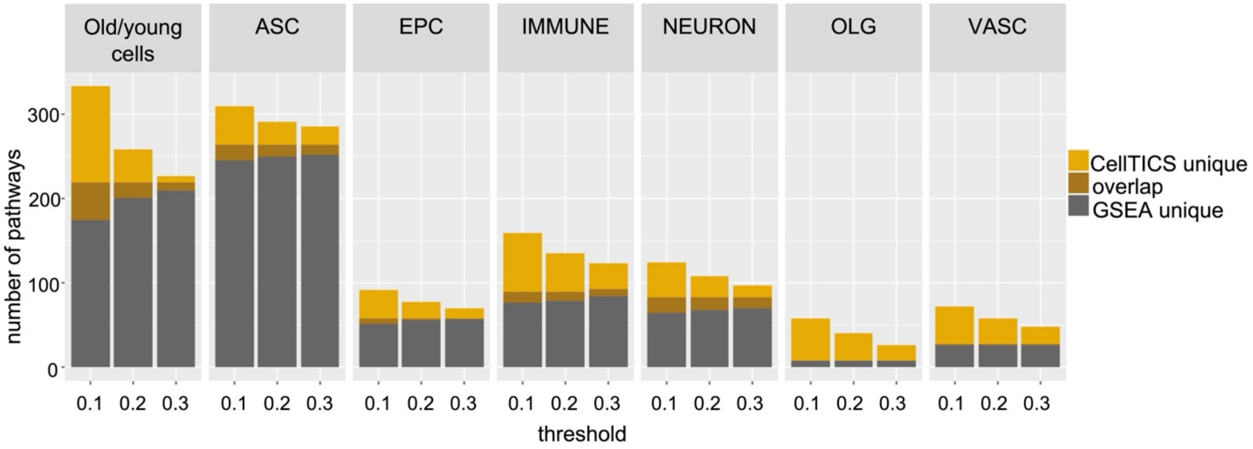

Supplementary Figure S6. Pathway identification of CellTICS with different D_p thresholds. Their overlaps with pathways identified by GSEA are also shown.

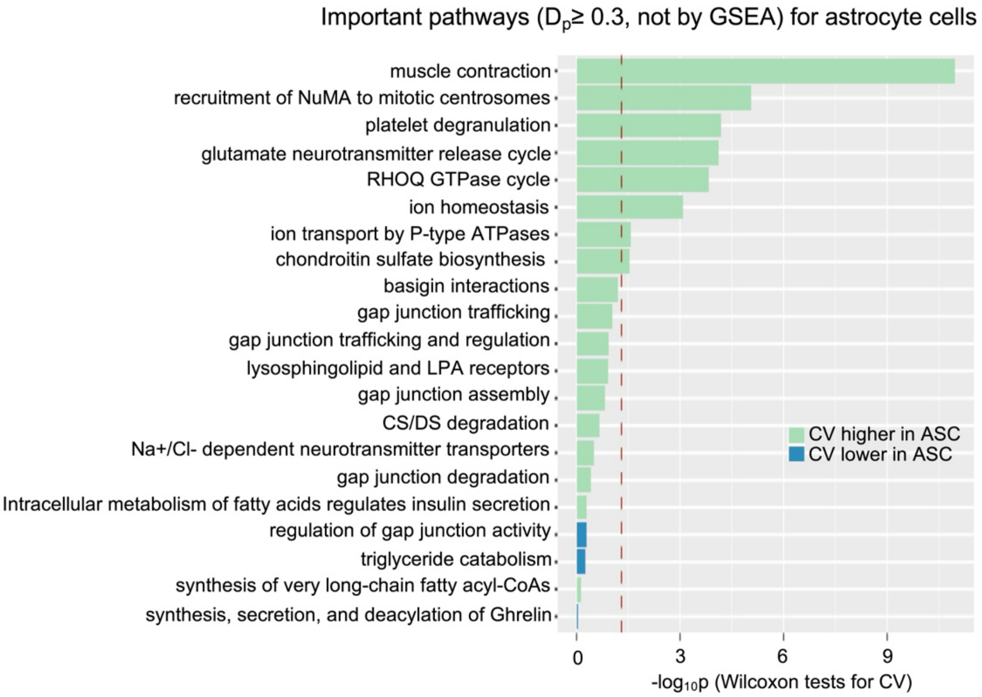

Supplementary Figure S7. Differential expression stochasticity of important pathways identified for astrocytes. Wilcoxon tests are performed to measure the differential expression stochasticity. The red dashed line marks the p-value of 0.05. The color of each pathway indicates whether the group exhibits a higher or lower coefficient of variation.

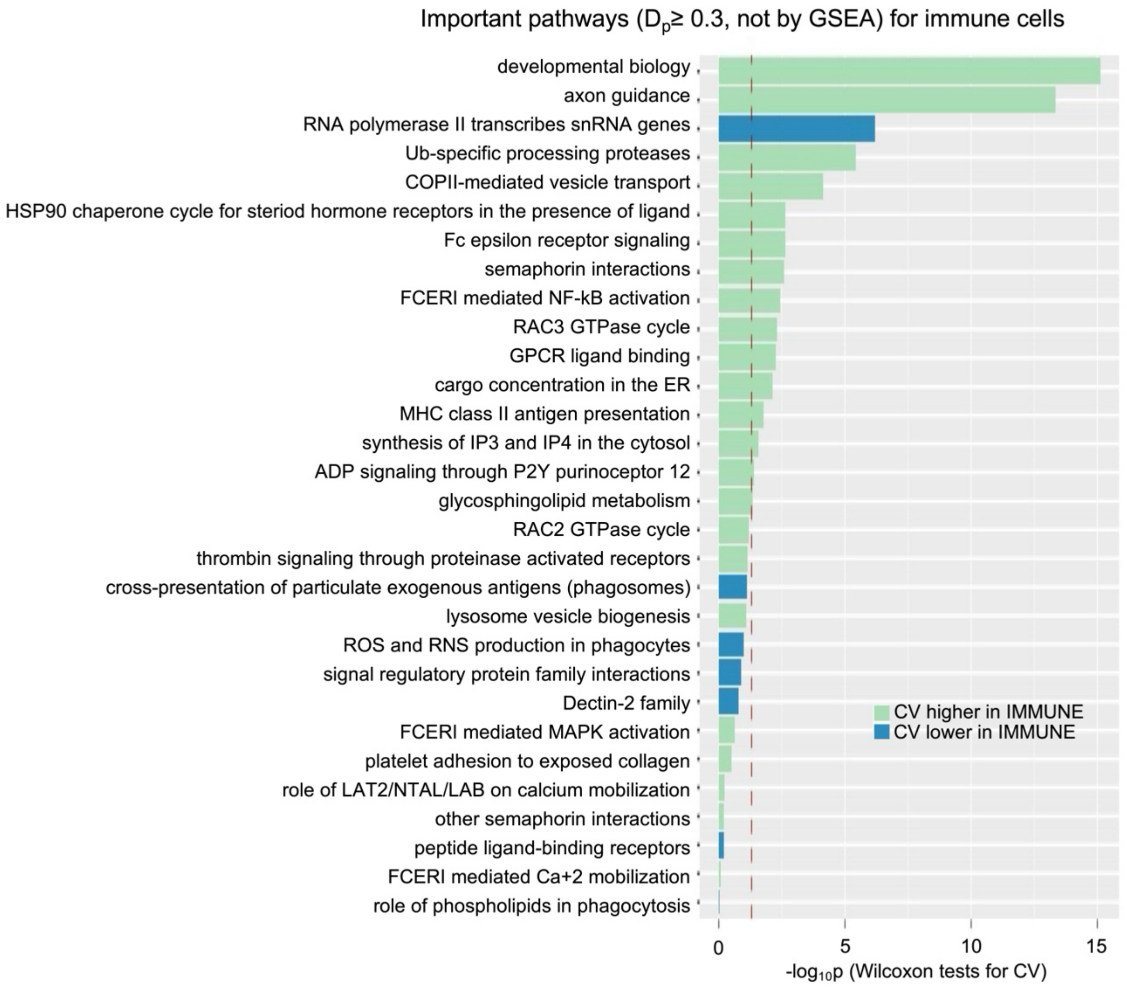

Supplementary Figure S8. Differential expression stochasticity of important pathways identified for immune cells. Wilcoxon tests are performed to measure the differential expression stochasticity. The red dashed line marks the p-value of 0.05. The color of each pathway indicates whether the group exhibits a higher or lower coefficient of variation.

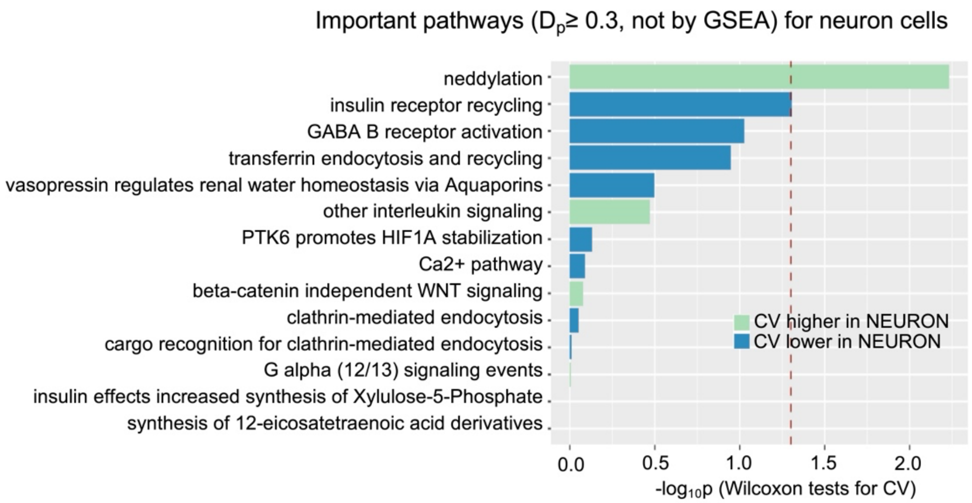

Supplementary Figure S9. Differential expression stochasticity of important pathways identified for neuron cells. Wilcoxon tests are performed to measure the differential expression stochasticity. The red dashed line marks the p-value of 0.05. The color of each pathway indicates whether the group exhibits a higher or lower coefficient of variation.

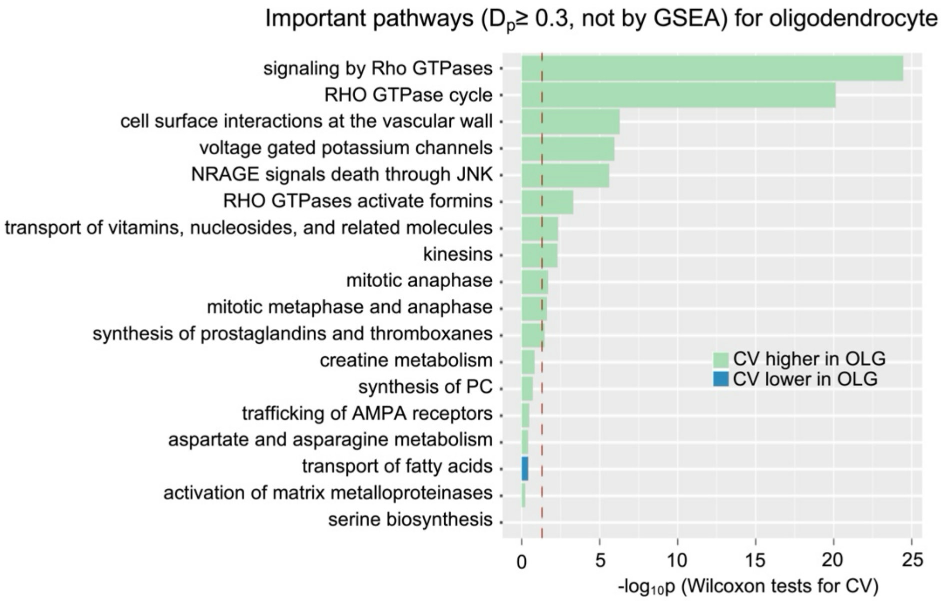

Supplementary Figure S10. Differential expression stochasticity of important pathways identified for oligodendrocytes. Wilcoxon tests are performed to measure the differential expression stochasticity. The red dashed line marks the p-value of 0.05. The color of each pathway indicates whether the group exhibits a higher or lower coefficient of variation.

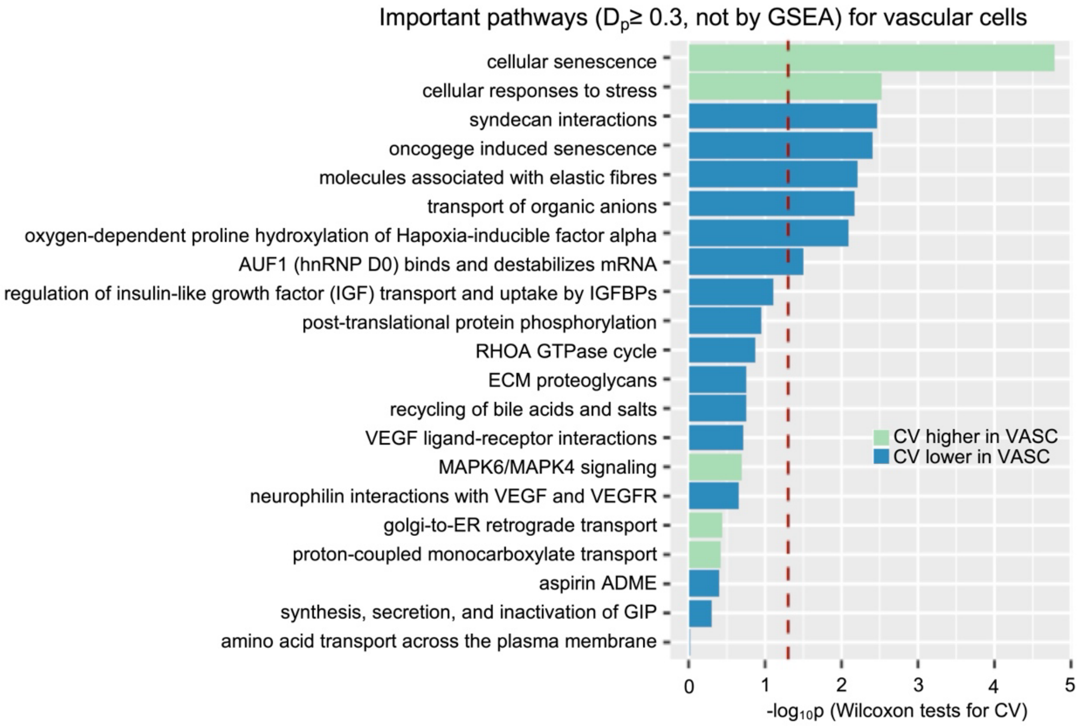

Supplementary Figure S11. Differential expression stochasticity of important pathways identified for vascular cells. Wilcoxon tests are performed to measure the differential expression stochasticity. The red dashed line marks the p-value of 0.05. The color of each pathway indicates whether the group exhibits a higher or lower coefficient of variation.

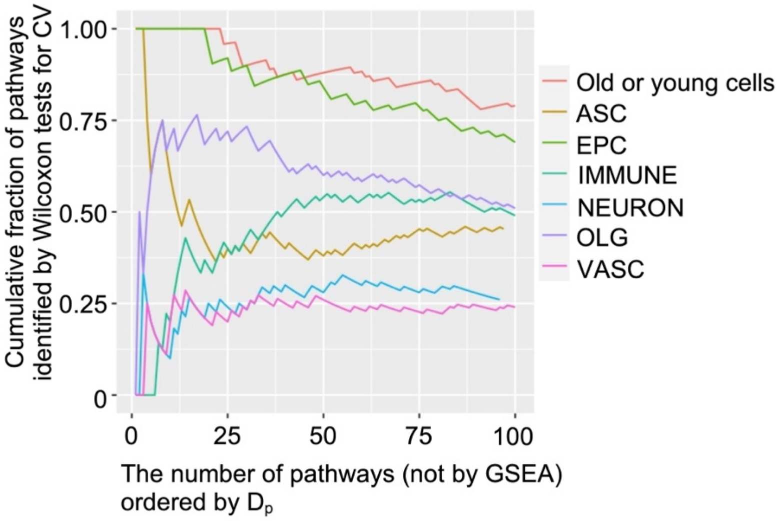

Supplementary Figure S12. Shared pathways between CellTICS and Wilcoxon tests on CVs. The cumulative fraction of pathways identified by Wilcoxon tests on CVs is plotted along pathways with decreasing D_p values in CellTICS. These pathways are not identified by GSEA.

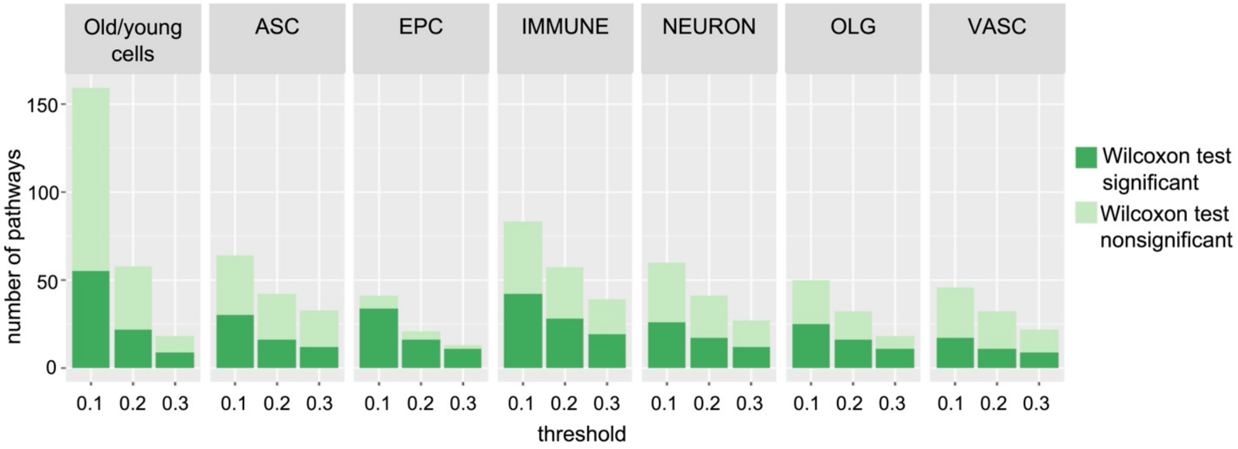

Supplementary Figure S13. Differential expression stochasticity of CellTICS-unique pathways. These pathways are identified by CellTICS with different D_p thresholds, and they are not identified by GSEA. Differential expression stochasticity is measured by the Wilcoxon test (p-value ≤ 0.05).

Supplementary Table S1. Information about the datasets used in CellTICS for predicting cell types or sub-cell types.

<table>
<tr><th>Dataset</th><th>Description</th><th>Number of cell types</th><th>Number of sub-cell types</th><th>Number of genes</th><th>Number of cells</th><th>Data file size</th><th>Link</th></tr>
<tr><td rowspan="2">L5MB</td><td rowspan="2">Level 5 adolescent mouse brain cells</td><td rowspan="2">7</td><td>Training set: 237</td><td rowspan="2">27998</td><td>Training set: 20731</td><td>1080 MB</td><td rowspan="2">http://mousebrain.org/</td></tr>
<tr><td>Test set: 220</td><td>Test set: 6811</td><td>365MB</td></tr>
<tr><td rowspan="2">DropViz HC</td><td rowspan="2">DropViz mouse brain hippocampus cells</td><td rowspan="2">11</td><td>Training set: 101</td><td rowspan="2">17874</td><td>Training set: 19347</td><td>661MB</td><td rowspan="4">http://dropviz.org/</td></tr>
<tr><td>Test set: 100</td><td>Test set: 6443</td><td>220MB</td></tr>
<tr><td rowspan="2">DropViz FC</td><td rowspan="2">DropViz mouse brain frontal cortex cells</td><td rowspan="2">9</td><td>Training set: 80</td><td rowspan="2">18533</td><td>Training set: 25157</td><td>891MB</td></tr>
<tr><td>Test set: 78</td><td>Test set: 8380</td><td>297MB</td></tr>
<tr><td rowspan="2">AMB whole</td><td rowspan="2">Aging mouse brain cells of all mice</td><td rowspan="2">6</td><td rowspan="2">12 (old and young for each cell type)</td><td rowspan="2">13669</td><td>Training set: 27453</td><td>987 MB</td><td rowspan="6">https://www.ncbi.nlm.nih.gov/geo/query/acc.cgi?acc=GSE129788</td></tr>
<tr><td>Test set: 9148</td><td>419 MB</td></tr>
<tr><td rowspan="2">AMB OY</td><td rowspan="2">Aging mouse brain cells, using old to predict young</td><td rowspan="2">6</td><td rowspan="2">25</td><td rowspan="2">13669</td><td>Old: 20746</td><td>950 MB</td></tr>
<tr><td>Young: 15855</td><td>632 MB</td></tr>
<tr><td rowspan="2">AMB YO</td><td rowspan="2">Aging mouse brain cells, using old to predict young</td><td rowspan="2">6</td><td rowspan="2">25</td><td rowspan="2">13669</td><td>Young: 15855</td><td>632 MB</td></tr>
<tr><td>Old: 20746</td><td>950 MB</td></tr>
<tr><td rowspan="2">PBMC 32</td><td rowspan="2">Peripheral blood mononuclear cells, using 10Xv3 to predict 10Xv2</td><td rowspan="2">3</td><td>10Xv3: 8</td><td>21905</td><td>10Xv3: 19690</td><td>650 MB</td><td rowspan="4">https://doi.org/10.5281/zenodo.3357167</td></tr>
<tr><td>10Xv2: 9</td><td>22280</td><td>10Xv2: 23154</td><td>772 MB</td></tr>
<tr><td rowspan="2">PBMC 2D</td><td rowspan="2">Peripheral blood mononuclear cells, using 10Xv2 to predict</td><td rowspan="2">3</td><td rowspan="2">9</td><td>22280</td><td rowspan="2">23154</td><td>772 MB</td></tr>
<tr><td>16480</td><td>730 MB</td></tr>
</table>

	Drop-seq						
PBMC D3	Peripheral blood mononuclear cells, using Drop-seq to predict 10Xv3	3	Drop-seq: 9	16480	Drop-seq: 23154	730 MB	
			10Xv3: 8	21905	10Xv3: 19690	650 MB	
ASD	Autism spectrum disorder human cells	11	22 (ASD and control for each cell type)	36501	Training set: 45641	4760 MB	https://cells.ucsc.edu/?ds=autism
					Test set: 15208	1580 MB	

Supplementary Table S2. Run time of CellTICS across all datasets.

**Dataset**	L5MB	DropViz HC	DropViz FC	AMB whole	AMB OY	AMB YO	PBMC 32	PBMC 2D	PBMC D3	ASD
**Run time(s)**	1413	1382	1559	1600	1291	1100	1223	1320	1324	4643
